# Ultrasound-Guided Synovial Biopsy Can Be Effectively and Safely Performed in Different Clinical Settings Favouring a Widespread Application of Precision Medicine in Rheumatoid Arthritis: A Post-hoc Analysis of Three Clinical Trials

**DOI:** 10.3390/jcm15062233

**Published:** 2026-03-15

**Authors:** Mattia Congia, Stefano Marini, Alessandra Nerviani, Felice Rivellese, Georgina Thorborn, Rebecca Hands, Maria Maddalena Angioni, Elisabetta Chessa, Alberto Floris, Piero Mascia, Matteo Piga, Frances Humby, Stefano Marcia, Costantino Pitzalis, Alberto Cauli

**Affiliations:** 1 Rheumatology Unit, Department of Medicine and Public Health, Azienda Ospedaliera Universitaria di Cagliari, University of Cagliari, 09124 Cagliari, Italy; 2 Radiology Unit, SS Trinità Hospital, ATS Sardegna, 09121 Cagliari, Italy; 3 Centre for Experimental Medicine and Rheumatology, William Harvey Research Institute, Barts and the London School of Medicine and Dentistry, Queen Mary University of London, London E1 2AT, UK

**Keywords:** rheumatoid arthritis, synovial biopsy, precision medicine

## Abstract

**Objectives**: In the perspective of an increasingly widespread application of precision medicine in rheumatoid arthritis (RA), this study aimed to compare efficacy and safety of ultrasound-guided synovial biopsy (US-SB) performed in an experienced rheumatology and community hospital setting. **Methods**: A post hoc analysis of R4RA, STRAP and STRAP-EU trials was performed, comparing US-SB performed in a radiology department of a community hospital without experience in RA (*n* = 14), versus a rheumatology academic centre with a high expertise in RA management and US-SB (*n* = 16). Suitability of specimens for histological and transcriptomic analysis (tissue and RNA quality) was analyzed as the main outcome. **Results**: Demographic and clinical features of the two patients’ groups were similar, except of disease duration (*p* < 0.05). No differences were recorded regarding site and ultrasound of the biopsied joint. Suitability for histological (% of gradable tissue) and transcriptomic analysis (RIN >3) was similar in the two cohorts (both 85.7% vs. 87.5%, *p* = 0.88). Proportion of gradable biopsies in total (59.2% vs. 59.5%, *p* = 0.96) and for each patient (52% vs. 56.15%, *p* = 0.77), were similar in both cohorts. Adverse events were rare (two in community hospital cohort, one in rheumatology cohort, *p* = 0.54), none considered severe. Seven patients in the community hospital experienced mild or severe pain, only two referred the same in the rheumatology cohort (*p* = 0.04). **Conclusions**: US-SB can be safely and effectively performed in a community hospital without experience in RA. A larger diffusion of this technique could allow to pursuit a tailored approach also in ordinary rheumatology outpatient clinics.

## 1. Introduction

Rheumatoid Arthritis (RA) therapy has dramatically changed in the last 20 years with the advent of new biological targeted therapeutics. Although their efficacy in reducing joint inflammation and subsequent damage has been proven by clinical trials and international registries, on an individual patient level, rheumatologists do not have any reliable tool to predict response to therapy and are forced to pursue a “try and see” prescribing strategy, as the aspiration of a precision medicine approach still remains unfulfilled. In this context, however, advanced multi-omic analyses of synovial tissue biopsies are bringing a new dimension not only in deepening understanding of RA pathogenesis but also as a possible source of prognostic biomarker and as a predictor of treatment response. Several ongoing biopsy-based trials are investigating the potential role of synovial tissue in patient stratification with promising results [1,2,3,4,5,6].

Despite the general impression among rheumatologists, synovial tissue is not difficult to obtain from RA patients. While arthroscopic synovial biopsy (SB) is considered the gold standard, as it is excellent in retrieving high-quality synovial tissue and RNA, with a very good safety profile, it has some major limitations due to costs and the need of dedicated expertise and facilities [7,8]. Ultrasound (US)-guided needle synovial biopsy (US-SB) is a relatively new technique that has been shown to be safe and well tolerated by patients [9,10,11]. Although US-SB allows only indirect visualization of synovial membrane, it does enable a precise retrieval of specimens suitable for histological and molecular analysis. Recently, Humby et al. compared four different synovial biopsy techniques (arthroscopic, blind needle (BN) biopsy, US-SB, and portal and forceps (P&F) US guided biopsy) in small and large joints of active RA patients and found that US-SB has performances similar to arthroscopic biopsy in terms of gradable tissue and RNA integrity and quality. The safety profile was found to be good with very few side effects and only minor discomfort reported by patients [12].

Despite this considerable progress, US-SB is not yet routinely available outside large rheumatology centres because of the lack of rheumatologists trained in the procedure, which hampers the feasibility of routine stratified precision medicine trials for a large number of RA patients. In community hospitals, Radiology departments usually perform US biopsy of deep organs such lungs, liver, or kidneys, therefore they should have all the facilities and capabilities needed to safely and effectively perform US-SB in RA patients. Moreover, US-SBs are increasingly being used by radiologists to diagnose periprosthetic joint infection or synovial tumours in orthopedic patients [13,14]. This could be an opportunity for small rheumatology centres to overcome this obstacle; favouring a widespread use of this simple procedure could lead to greater implementation of precision medicine studies outside highly specialized centres. In almost all studies conducted to date, SB were executed by rheumatologists in high specialized centres and scarce data are available on the US-guided technique carried out in different clinical settings and by different specialists (i.e., radiologists), without known experience in RA, to include the retrieval of synovial tissue suitable for pathobiological analyses [15,16].

Therefore, the aim of the present study is to compare the performance, in terms of safety and effectiveness, of US-SB performed by skilled rheumatologists in a large university hospital with US-SB performed in a general hospital with non-specific experience in inflammatory joint disease.

## 2. Materials and Methods

### 2.1. Patients

This is a cross-sectional post hoc analysis of 30 patients recruited to three trials: “A Randomised, open labelled study in anti-Tumor Necrosis Factor alfa (TNFa) inadequate responders to investigate the mechanisms for Response—Resistance to Rituximab versus Tocilizumab in RA” (R4RA, EudraCT number: 2012-002535-28), “Stratification of Biologic Therapies for RA by Pathobiology” (STRAP, EudraCT number: 2014-003529-16) and “STRAP-EU” (EudraCT number: 2017-004079-30) [3,4]. The former recruited conventional synthetic modifying anti rheumatic drugs (csDMARDs) inadequate responders, while the latter included anti-TNF inadequate responders RA patients. Inclusion and exclusion criteria were similar for those trials, and are available in the respective publications. Briefly, patients diagnosed with RA according to 2010 American College of Rheumatology (ACR)/European League against Rheumatism (EULAR) classification criteria [17] with active disease and indication for biological therapy according to National Institute for Health and Care Excellence (NICE) guidelines, underwent US-SB of a clinically active joint prior to initiation of a new treatment. Synovial samples were analysed trough immunohistochemistry (IHC) and transcriptomics to define different subtypes of RA and predict drug response [3,4]. Both studies were authorized by the local ethic committee (Comitato Etico Indipendente Azienda Ospedaliera Universitaria di Cagliari, PG/2015/11543; PG2018/9999), and all patients included signed informed consent before enrolment. The aforementioned studies were conducted in compliance with the Declaration of Helsinki, International Conference on Harmonisation Guidelines for Good Clinical Practice, and local country regulations. Ethical review and approval were waived for this study due to the fact that this secondary analysis was carried out using only previously collected anonymized data for which patients had already provided consent. It did not require new experiments and it does not introduce new burdens or risks to participants and remains consistent with what they were informed about at the time of consent.

For the purpose of this sub-analysis, patients were divided into two cohorts according to the clinical setting where US-SB was performed. All the patients followed at Rheumatology Unit of University Hospital of Cagliari, Italy, were biopsied from January 2016 to June 2019 at the Radiology Department, SS Trinità Hospital in Cagliari following a three-day appropriate training (community hospital cohort). Patients enrolled at Barts and the London School of Medicine, Queen Mary University of London, UK with strong experience in US-SB technique were biopsied and served as controls (rheumatology cohort). All the biopsies were performed using the US-SB technique as previously described [9]. Control patients were selected according to age, sex, disease features, biopsy site and time (month/year). At least six samples were retrieved in each biopsy procedure from different sites of the same joint.

Baseline demographics including disease duration, disease activity, seropositivity for rheumatoid factor (RF) and anti-citrullinated protein antibodies (ACPA) were collected. US scan of hands and wrists was routinely performed at biopsy visit in case of a biopsy in a different site (e.g., knee or elbow); US scan of the selected joint was also performed. Gray-scale (GS) and power Doppler (PD) were evaluated with a semi quantitative scale (0–3) as previously described [9].

### 2.2. Synovial Histopathological Assessment

Synovial samples were included in paraffin and cut at 3 µm thickness for haematoxylin and eosin and IHC staining. Stained slides were examined, and the following parameters collected: number of patients with gradable samples, number of graded synovial fragments in total and per patient, as previously described [12]. Synovitis score was assessed with Krenn Score [18].

### 2.3. RNA Extraction

RNA extraction was performed as described extensively previously [19]. Briefly, synovial samples were homogenized in TRIzol reagent (ThermoFisher Scientific, Life Technologies, Invitrogen Division, Cheshire, UK), according to manufacturer recommendations, and subsequently RNA was extracted with phenol-chloroform and centrifugated with ice cold isopropanol. RNA pellet was then resuspended in RNAse-free water. RNA concentration was measured with a NanoDrop 2000 spectrophotometer (LabTech, Rotheram, UK) and RNA quality (RNA integrity number, RIN) was measured with an Agilent 2100 Bioanalyzer or Agilent TapeStationSystem (Agilent, Santa Clara, CA, USA). Reference values for RIN range from 1 (completely degraded) to 10 (intact). Samples with good quality (RIN > 3) were considered suitable for subsequent transcriptomic analysis [20,21].

Histopatological assessment and RNA extraction were performed in the same laboratory for all the samples of this study (Experimental Medicine and Rheumatology, William Harvey Research Institute, Queen Mary University of London, London, UK).

### 2.4. Adverse Events

Adverse events were recorded during the procedure and at the following visit scheduled as per protocol of the original studies. A set of adverse events of special interest was identified and included: hemarthrosis, wound and/or joint infection, deep venous thrombosis, neurological damage, tendon/ligament damage, syncope/pre-syncope, and arthralgia. Subjective assessment of discomfort during biopsy was collected using a 5-point scale questionnaire: no discomfort, mild discomfort, moderate discomfort, mild pain, severe pain.

### 2.5. Statistical Analysis

Demographic features of the patients were described with mean (±standard deviation) or median (interquartile range), when appropriate. Qualitative variables were described as relative frequencies. Chi-square or Fisher tests were applied for qualitative variables, whether Student’s, Mann–Whitney or Kruskal–Wallis tests were used for quantitative variables.

## 3. Results

### 3.1. Patients

Fourteen patients were enrolled at the Rheumatology Unit of Cagliari University Hospital (community hospital cohort) and 16 matched patients were selected from Barts and The London School of Medicine (rheumatology cohort). Demographics and disease specific features are shown in Table 1.

No statistical differences were noted between the two cohorts, except for disease duration (Rheumatology cohort 4.26 ± 3.95 years vs. community hospital 8.8 ± 4.4 years, *p* 0.002). All patients, as expected according to respective protocols of the two studies, experienced previous treatment with csDMARDS and/or biologic DMARDs and had high disease activity according to Disease Activity Score 28 (DAS28) index (>5.1).

Biopsy site and outcomes are shown in Table 2.

From the community hospital cohort, three (21%) biopsies were performed in the knees, seven (50%) in the hands (6 metacarpophalangeal, MCP; one proximal interphalangeal PIP) and four (29%) in the wrists. In the Rheumatology Cohort, 10 biopsies were performed in the wrists (63%), three (19%) in the hands (3 MCP) and three in the knees (19%). No statistical significant difference was noted in biopsy site between the two centres, though in the rheumatology cohort there were more wrist biopsies and fewer MCP and PIP biopsies. Ultrasound of the biopsied joint revealed similar synovial thickness for both cohorts: community hospital cohort 2.5 (2–3), community hospital cohort 2 (2–3); *p* = 0.59, and power Doppler score, community hospital cohort 2 (1–2), Rheumatology cohort 2 (1.75–2); *p* = 0.87.

### 3.2. Histological Analysis

Out of 14 patients in the community hospital cohort, two subjects were not gradable for histological analysis due to very poor quality, while in the Rheumatology cohort two out of 16 were judged ungradable (14.3% vs. 12.5%; *p* = 0.88). Reciprocally this meant that synovial tissue suitable for IHC analysis was successfully obtained in the large majority of patients: 85.7% in the community hospital cohort and 87.5% in the rheumatology cohort. Even when the analysis was performed stratifying for joint size (small joints = MCP, PIP; large joints = knee, wrist), it was not possible to observe any statistically significant difference between the two cohorts. We next analyzed the proportion of fragments passing quality control in the two cohorts. In the community hospital cohort 76 tissue biopsies were collected in total from all the patients and 45 were gradable (59.2%). The mean value of gradable pieces for each patient was 52% (±30.2). The overall quality of the synovial samples remained similar during the whole duration of the trial, indicating that good synovial biopsies were performed from the beginning. In the Rheumatology cohort 126 pieces of tissue were retrieved, and 75 were gradable (59.5%, vs. community hospital *p* = 0.96). In this whole cohort the mean value of gradable pieces for each patient was 56.15% (±49.1) (vs community hospital cohort *p* = 0.77).

### 3.3. RNA Extraction

Total extracted RNA was similar between the two cohorts: in the community hospital cohort the mean RNA quantity was 0.72 (±0.75) µg, while in the Rheumatology cohort was 1.16 (±0.57) (*p*: 0.25). RNA quality was slightly better in community hospital cohort (mean RIN 7.05 (±7.1) vs. 5.67 (±5.7); *p* = 0.01), but in both cohorts a similar proportion of patients was suitable for transcriptomic analysis, (community hospital cohort 12/14 (85.7%) vs. Rheumatology cohort 14/16 (85%); *p* = 0.88). Results of the transcrptomic analysis of the two trials have recently been published [22,23].

### 3.4. Adverse Events

As expected, US-SB was generally well tolerated. Nine patients in total experienced mild or severe pain during procedure (seven in the community hospital cohort, two in the Rheumatology cohort; *p* = 0.04). Two patients in the community cohort experienced adverse events (one fainting during the procedure, one worsening of arthralgia of the biopsied joint reported at the visit after the biopsy). One adverse event was reported in the rheumatology cohort (worsening of pain in the biopsied joint reported at the visit after the biopsy; *p* = 0.54), with no serious adverse events reported in both cohorts.

## 4. Discussion

In this cross-sectional study, we demonstrated that US-SB could be safely and effectively performed in a community hospital by interventional radiologists after appropriate training. This could be an important step towards the extension of the use of US-SB, also in smaller peripheral centres for both clinical and research purposes. Very few reports have been published on the performance of US-SB executed outside specialized centres and none in the context of a randomized clinical trial in RA. Sitt et al. reported a retrospective case series of 111 US-SBs, yielding a very high success rate: only in four biopsies was synovial tissue not retrieved (success rate 96.3%). It must be noted that the indication for SB was exclusion of joint tumour or infection, and no specific analyses were performed or reported to confirm a diagnosis other than those. For those without evidence of joint tumour or infection, diagnosis was based on clinical picture and lab test. In the end, seven patients were diagnosed with RA, one with overlap SLE/RA and 37 with other rheumatological conditions, but success rates for this specific population was not reported. SB was reported to be well tolerated with only one patient reporting transient vaso-vagal attack [16]. Marin et al. reported a similar success rate for synovial tissue retrieval in their study (94%), involving 83 patients with monoarthritis. Also in this study, SB was very well tolerated, with no adverse events reported [15].

Our data are concordant with these reports: in both the community hospital and rheumatology cohorts, we had a similar high success rate, both for synovial tissue suitable for IHC analysis and for RNA extraction (85.7% in community hospital cohort and 87.5% in rheumatology cohort). It is important to stress that in the community hospital cohort, good quality samples were retrieved from the beginning of the trials, with no apparent “learning curve”. This indicates that radiologists who do not have specific expertise in inflammatory joint disease, but a longstanding experience in other organ biopsies, can perform SB effectively with a short period of training.

In our study we reported a very small number of adverse events in both cohorts and, notably, none of them were serious or permanent. This is concordant with published studies and provides more evidence that interventional radiologists can safely perform US-SB.

The utility of SB for the prediction of disease course and treatment response in RA is a matter of debate, but emerging evidence indicates that SB can effectively be used to stratify patients according to disease severity or drug response [21]. In the Pathobiology of Early Arthritis Cohort (PEAC), 144 early RA patients were biopsied before starting csDMARDs treatment: patients with synovial evidence of B-cell infiltration had a higher disease activity, expressed as DAS28, and a higher baseline erosive burden. Furthermore, those patients had a greater increase in erosive score after 1 year of treatment [1]. Increasing the possibility of drug response is crucial in RA as uncontrolled disease activity leads to irreversible joint damage, and a tailored therapy, with the support of synovial biomarkers, could improve the short- and long-term outcomes of RA patients. The results of R4RA and STRAP/STRAP-EU were published in the last two years. Humby et al., in the first trial, showed that antiTNF failure patients with low or absent B-cell lineage synovial transcripts expression had significantly higher response rates to tocilizumab compared to rituximab [3]; namely, twice and four times as many patients reached primary end-point (CDAI > 50% from baseline) or CDAI major treatment response (CDAI > 50% and CDAI < 10.1) respectively in the tocilizumab-treated group compared to rituximab. Those promising results were not confirmed in STRAP/STRAP-EU trials involving biologic naïve RA patients, where the low or absent B-cell lineage synovial transcripts expression did not predict treatment efficacy with rituximab versus other biological drugs (etanercept and tocilizumab grouped together), even though some secondary outcomes (including DAS28 remission at week 16) were achieved. These results highlight the need for a more complex approach to identify synovial biomarkers useful in predicting drug response in this population [4].

Our study has several strengths, being the first to directly compare two different specialties in performing SB in the context of a clinical trial, with standardized evaluations and procedures, and it has specific outcomes of success (tissue eligibility for IHC classification and RNA extraction). Furthermore, patients involved are representative of different stages of RA, including different therapies and different joint involvement, with different sites being biopsied. Additionally, we specifically focused on a specific set of immediate adverse events, which are of major concern for physicians that perform SB, providing further evidence of the safety of US-SB. The favourable risk profile is reassuring for other specialists without experience in SB that may perform US-SB in their clinical practice. Lastly, although conducted before 2021, the two analyzed trials comply with the recently published “EULAR Points to consider for minimal reporting requirements in synovial tissue research”, which define the “gold standard” for synovial histopathology research in chronic arthritis [11].

Our study also has some limitations. The relatively small number of patients and being a post hoc analysis limit the statistical power and external validity of these findings, though the standardization of the procedures in the context of a multi-centric randomized clinical trial provides very high-quality data. It was not possible to increase the sample size of this analysis, since only our centre had the support of an external interventional radiology department, while all the other centres involved in the trials performed US-SB in a highly specialized rheumatological academic environment, with an in-depth experience of US-SB.

## 5. Conclusions

In conclusion, our findings suggest that US-SB could be safely and effectively performed by interventional radiologists, without long-standing experience in rheumatological diseases, though future prospective studies could be useful to give more robust evidence and also allow a broader range of comparisons. This can open opportunities for small rheumatology centres to participate in future biopsy-driven stratified medicine trials investigating whether molecular pathology, similarly, for example, to cancer therapy, has clinical utility in determining drug allocation of highly selective biologic therapies to patients with high expression levels of the target in the disease tissue towards the implementation of a personalized medicine approach.

## Figures and Tables

**Table 1:** Demographic and clinical features of the two cohorts.

	Interventional Radiology Cohort (*n* = 14)	Rheumatology Cohort (*n* = 16)	*p* Value
Age (years)	55 ± 11	51.4 ± 12.5	0.374
Female Sex	10 (71.4%)	13 (81.25%)	0.40
Disease duration (years)	8.8 ± 4.7	4.26 ± 3.95	0.002
ACPA+	13 (92.8%)	11/15 (73.3%)	0.16
RF+	12 (85.7%)	11 (68.5)	0.27
Biologic treatment ever	4 (28.6%)	8 (50%)	0.23
TNFi	4	8	
ABA	1	0	
csDMARDs treatment ever	14 (100%)	16 (100%)	1
HCQ	10	9	
MTX	11	14	
LEF	5	2	
SSZ	1	9	
GS	1	0	
CyA	0	1	
DAS28 > 5.1	14 (100%)	16 (100%)	1

ACPA: anti citrullinated proteins antibodies, RF: rheumatoid factor, “+” indicates positive; TNFi: tumour necrosis factor inhibitors. HCQ: hydroxychloroquine, MTX: methotrexate, LEF: leflunomide, SSZ: sulfasalazine, GS: gold salts, CyA: cyclosporine, ABA: abatacept.

**Table 2:** Biopsy site and outcomes.

Biopsy Site	Radiology	Rheumatology	*p* Value
Knee	3 (21.4%)	3 (18.8)	0.85
MCP-PIP	7 (50%)	3 (18.8)	0.07
Wrist	4 (28.6%)	10 (62.5)	0.06
**US**			
Synovial Thickness grade	2.5 (2–3)	2 (2–3)	0.59
Power-Doppler grade	2 (1–2)	2 (1.75–2)	0.87
**Biopsy quality** **(Histologic Analysis)**			
Very poor	2 (14.2%)	2 (12.5%)	0.88
Poor	3 (21.4%)	2 (12.5%)	0.51
Moderate	5 (35.7%)	2 (12.5%)	0.13
Good	4 (28.6%)	6 (37.5%)	0.60
Excellent	0	4 (25%)	0.10
Suitable (≥poor)	12 (85.7%)	14 (87.5%)	0.88
Suitable (Small Joints)	7/7	3/3	1
Suitable (Large Joints)	5/7	11/13	0.58
Krenn Score	5 (5–6)	3 (2.3–5.3)	0.41
**RNA extraction**			
Total RNA (µg)	0.84 (0.33–1.15)	0.67 (0.38–1.8)	0.81
RIN	7.05 (±7.1)	5.67 (±5.7)	0.01
RIN > 3	12 (85.7%)	14 (87.5%)	0.88
Adverse events			
Minor	2 (14.2%)	1 (7%)	0.54
Severe	0	0	

MCP: metacarpophalangeal, PIP: proximal interphalangeal, RIN: RNA integrity number.

## Data Availability

Data are available upon reasonable request.

## Abbreviations

The following abbreviations are used in this manuscript:

RA	Rheumatoid Arthtiris
US	Ultrasound
US-SB	Ultrasound guided synovial biopsy
SB	Synovial Biopsy
BN	Blind Needle
P&F	Portal and Forceps
TNFa	Tumour Necrosis Factor Alfa
R4RA	A Randomized, open labelled study in anti-TNFa inadequate responders to investigate the mechanisms for Response—Resistance to Rituximab versus Tocilizumab in RA
STRAP	Stratification of Biologic Therapies for RA by Pathobiology
csDMARDs	Conventional synthetic modifying anti rheumatic drugs
ACR	American College of Rheumatology
EULAR	European League against Rheumatism
NICE	National Institute for Health and Care Excellence
IHC	immunohistochemistry
RF	rheumatoid factor
ACPA	anti-citrullinated protein antibodies
GS	Gray-scale
PD	Power Doppler
RIN	RNA integrity number
TNF-i	tumour necrosis factor inhibitors
HCQ	hydroxychloroquine
MTX	Methotrexate
LEF	Leflunomide
SSZ	Sulfasalazine
GS	gold salts
CyA	Cyclosporine
ABA	Abatacept
DAS28	Disease Activity Score 28
MCP	metacarpophalangeal
PIP	proximal interphalangeal
PEAC	Pathobiology of Early Arthritis Cohort
